# Newly Isolated *Vibrio cholerae* Non-O1, Non-O139 Phages

**DOI:** 10.3201/eid1004.030413

**Published:** 2004-04

**Authors:** B. L. Sarkar, Amar N. Ghosh, Anindito Sen, D. P. Rodrigues

**Affiliations:** * National Institute of Cholera & Enteric Diseases (ICMR) WHO Collaborating Centre for Research and Training on Diarrheal Diseases, Kolkata, India; † Instituto Oswaldo Cruz, Rio de Janerio, Brazil

**Keywords:** *Vibrio cholerae*, Electron Microscopy, Enteric

**To the Editor:** The epidemic cholera caused by *Vibrio cholerae* O1 appeared in Latin America in 1991 after a 100-year absence. Following its explosive appearance in Peru, travelers on the Amazon River brought cholera to Brazil by April 1991. It spread southward along the Atlantic Coast of Brazil, reaching Rio de Janeiro in February 1993.

Phage typing is a useful tool for studying the source or origin of *V. cholerae* for epidemiologic importance. Because of limitations of the Basu and Mukerjee scheme, a new phage-typing scheme for *V. cholerae* O1 was developed at the National Institute of Cholera and Enteric Diseases, India (*1*–*3*). During the course of a comprehensive study on the phage typing of *V. cholerae* O1, most strains isolated in Brazil were found to be sensitive with a set of 10 El Tor phages (ATCC 51352-B1–B10) (*4*). This finding prompted us to explore or ascertain the natural habitat of *V. cholerae* and cholera phages, if any, in an environmental reservoir in Brazil, particularly in Rio de Janeiro.

Samples were collected from selected points where sewage water connected with the main canal receives domestic and industrial effluents from the city on its way to the Atlantic Ocean. Two major environmental parameters, temperature and pH, were recorded at each station during collection. Samples were processed for phage isolation according to the procedure adopted for *V. cholerae* phages (*5*,*6*). Portions of the samples were also analyzed for enteric pathogens. A total of 32 sewage samples were collected in 8 months; 12 were isolated from Rio de Janeiro, 10 from the Amazon region in the North, and 10 from northeastern Brazil.

Each sample was divided into two parts; one section was used to isolate O1 phages using MAK 757 (ATCC 51352) as a propagating strain. The other portion was used for non-O1 phages; the non-O1 *V. cholerae* strain was used for propagation starting from O2 to O139. The procedure was repeated on nutrient agar for the appearance of plaques. The phages were purified from a single discrete plaque by the soft agar (0.8%) overlay method with the propagating strain of *V. cholerae* non-O1 (O2 to O139) until homogenous plaques were obtained. Phage lysates were prepared in nutrient broth (Difco Laboratories, Detroit, MI) with the propagating strain *V. cholerae* (both O1 and non-O1).

High-titer phages were obtained by the plate lysis procedure by using the agar overlay method (*5*) with multiplicity of infection 0.01 at an incubation temperature of 37°C. Concentration, purification, and electron microscopy study of these phages were performed as described by Ghosh et al. (1989) (*7*), using a FEI Tecnai 12 Bio Twin transmission electron microscope (FEI Europe BV, Eindhoven, Holland, the Netherlands). Measurements were made with Analysis (SIS GmbH, Munster, Germany) software. The homogeneity of each phage was studied by plaque morphology on nutrient agar following 10-fold serial dilution. Each high-titer phage (10^8^–10^9^ pfu/mL) was then used to determine routine test dilution (RTD) by the soft agar overlay method. In this experiment, the RTD used was the highest dilution that failed to give confluent or complete lysis. A variety of enteropathogens were included for susceptibility against these phages. A single colony from nutrient agar plate was injected into 5 mL of nutrient broth and incubated 2–3 h under stationary conditions at 37°C. A bacterial lawn was made on nutrient agar with this broth culture mixed with 3.5 mL of molten soft agar. The phage at RTD was spotted onto the plate and incubated at 37°C for 18 h. The next morning, the appearance of the zone of lysis was recorded. *V. cholerae* MAK 757 (ATCC 51352) was included as a positive control.

Of the 32 samples examined, two non-O1 phages (O6 and O34) sensitive to *V. cholerae* O6 and O34 were isolated from the same site, the Sarapui River in Rio. The plaques of both the phages were observed as clear and round, with a diameter of 3 to 4 mm. A total of 107 strains of *Vibrio* and *Enterobacteriaceae* were tested against these two phages. All strains were untypeable, except for the O6 and O34 serotypes, which were lysed by the phages specific for O6 (DR1) and O34 (DR2). During the study period, the recorded temperature was 25°C–38ºC, and pH ranged from 8 to 10. No correlation was observed with these two parameters and the isolation of phages.

**Figure 1:**
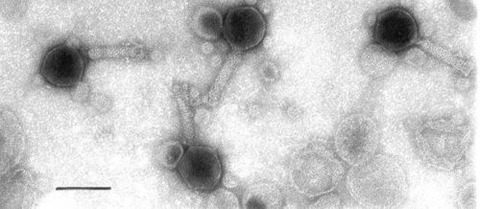
Electron micrograph of O34 vibriophage. Bar represents 100 nm.

**Figure 2:**
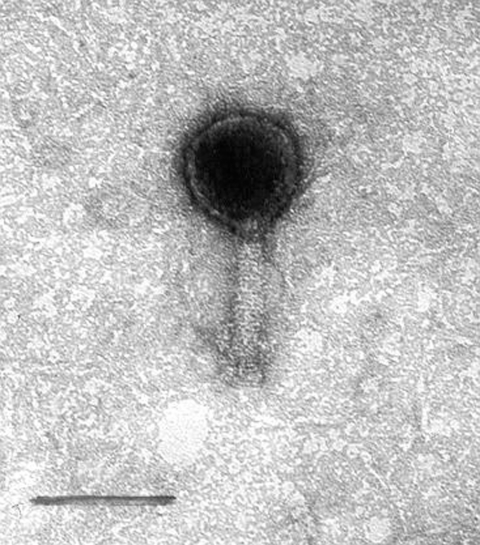
Electron micrograph of O6 vibriophage. Bar represents 100 nm.

The morphology of the phages was studied by negative staining electron microscopy. The O34 phages have a hexagonal head with a long tail. The diameter (distance between opposite apices) of the head is 83.0 ± 0.3 nm, while the length and width of the tail are 111.0 ± 0.8 nm and 17.0 ± 0.5 nm, respectively (Figure 1). The O6 phage has a similar form, with a head diameter of 77.5 ± 0.3 nm and a long tail 100.0 ± 0.6 nm in length and 19.0 ± 0.4 nm in width (Figure 2). The other enteropathogens isolated from the sewage samples were enteropathogenic *Escherichia coli*, *Salmonella* spp., and *Shigella* spp. (data not shown).

The use of vibriophages as tools for studying the source or origin of *V. cholerae* has contributed greatly to the understanding of the epidemiology of this disease. The emergence of *V. cholerae* O1 in South America in 1991 provoked major social and economic damage. A total of 60,000 clinical cases were reported from 1990 to 1996 in the coastal city of Rio de Janeiro. In 1997, approximately 2,600 confirmed cases of cholera were reported. In 1998, the number of cholera cases was 376. In 1999, the number of cases increased to 4,142; in 2000, the number decreased to 821 cases. This extreme variation in cholera cases continued during 2001, 2002, and 2003 (until February): the number of cholera cases reported were 665, 174, and 10, respectively. However, since 1993, no cholera cases caused by O1 have been reported. Only cases of non-O1 have been encountered, with O6 and O34 the predominant serotypes. A nationwide survey conducted by the National Reference Center for Cholera under Instituto Oswaldo Cruz is ongoing to isolate more phages in Brazil and neighboring countries.

To date, serotyping is the only identification tool for the characterization of non-O1 strains of *V. cholerae* (*8*). However, serotyping is only performed at a limited number of laboratories. For this study, all isolates from Brazil were sent to laboratories outside the country for serotyping. This step was expensive and time-consuming and posed risks during transit.

An alternative method is the use of phages for identifying non-O1 strains. This method offers an affordable monitoring system in less-developed countries such as Brazil. Phage O6 and O34 should at the least be useful for confirming the diagnosis of *V. cholerae* O6 and O34 infection and for differentiating *V. cholerae* O1 and non-O1 strains.
